# Synergistic Effect of Hyperglycemia and p27^kip1^ Suppression on Adult Mouse Islet Beta Cell Replication

**DOI:** 10.1155/2012/417390

**Published:** 2012-03-12

**Authors:** Szu-Tah Chen, Shin-Huei Fu, Samuel Hsu, Yu-Yao Huang, Brend Ray-Sea Hsu

**Affiliations:** ^1^Division of Endocrinology and Metabolism, Department of Internal Medicine, Chang-Gung Medical Center and Chang-Gung University, Taoyuan 333, Taiwan; ^2^Taiwan International Graduate Program, Graduate Institute of Life Science, National Defense Medical Center and Academia Sinica, Taipei 115, Taiwan

## Abstract

The complementary role of hyperglycemia and p27^kip1^ suppression on islet beta cell regeneration was investigated in a syngeneic mouse model. p27^kip1^ gene silencing was performed by infecting islets of C57BL/6 with shRNA lentiviral particles. At 54 hours after viral infection, p27^kip1^ protein content in cultured targeting islets was 22% of that in freshly isolated islets. Six days after transplantation to diabetic mice, targeting islet graft had considerably more cells with Ki67-staining nuclei than nontargeting islets. The mice in the targeting-islet group had a significantly shorter duration of temporary hyperglycaemia than mice in the non-targeting-islet group. The long-term *ex vivo* beneficial effect of p27^kip1^ silencing on graft function was also indicated by the significantly higher cumulative cure rate for diabetes in mice receiving 200 targeting islets than that in mice receiving 200 non-targeting islets. Our data suggest that hyperglycemia and persistent p27^kip1^ suppression have a synergistic effect on islet beta cell replication in adult mice.

## 1. Introduction

Blood glucose homeostasis can be disturbed by many clinical conditions such as pregnancy and obesity and cause *β*-cell mass expansion to meet the increased demand of insulin [1, 2]. However, the mechanism of action underlying the expansion of *β*-cells is currently unclear [3–6]. Although glucose has been postulated to regulate cyclin D2 in pancreatic islet beta cells and play a dominant role in beta cell compensation, it is not yet clear how glucose controls cell cycle of islet beta cells [7–10]. A previous study showed that the suppression of both cdk-inhibitors p27^kip1^ and p18^INK4c^, but not p27^Kip1^ alone, promotes endocrine tumour formation in rodents [11, 12]. Similarly, although cyclin D is important for cell proliferation, its overexpression does not trigger beta cell replication [13]. It suggests that adult islets are under multipoints control to regulate cell cycles for beta cell replication.

Our previous study revealed that primary islets of adult mice respond to chemical-mechanical digestion and purification procedures by markedly increasing cyclin B1 but reducing p27^Kip1^ protein level [14]. During the following 7 days of cultivation, maintenance of high level of cyclin B1 but rapid restoration of p27^Kip1^ of islets cultured in medium containing high glucose was noted [14]. The rapid restoration of p27^kip1^ level of cultured islets may explain the reason why cell number did not increase for fetal rat islet beta cells that are cultured in a medium containing 200 mg/dL glucose for 7 days [15]. Since the p27^Kip1^ is an important G1/G0 checkpoint for the progression of cell cycle, it is interesting to study whether persistent suppression of p27^kip1^ can enhance islet beta cell proliferation in a hyperglycaemic milieu. Since data obtained from experiments using cell lines, fetal islets or islets of knock-out mice to elucidate the role of p27^kip1^ and glucose on beta cell replication may not be suitable for applying to adult islets, in this study, we used primary islets of adult mice to investigate the complementary role of hyperglycemia and persistent p27^kip1^ suppression on beta cell regeneration.

## 2. Materials and Methods

Chemicals including Tris(hydroxyl-methyl)-aminomethane (Tris), histopaque-1077, type XI collagenase, and antibody against *β*-actin (AC-15) were obtained from the Sigma Chemical Company (St. Louis, Mo, USA). RPMI-1640 medium was purchased from GIBCO Invitrogen, Life Technologies, Inc. (Grand Island, NY, USA). Antibodies against cyclin B1 (GNS1), cyclin D1 (DCS-6), and p27^kip1^ (DCS-72.F6) were obtained from Thermo Fisher Scientific (Fremont, CA, USA), and antibody against FoxM1 (4G3) was purchased from Millipore Corporation (Bilerica, Mass, USA). Rabbit against mouse Ki67 polyclonal antiserum (ab15580) was purchased from Abcam Inc. (Cambridge, MA, USA).

### 2.1. Animal Care and Induction of Diabetes

 Male C57BL/6 mice (8–12 weeks old) were obtained from a local breeder and were administered a single intraperitoneal injection of streptozotocin 200 mg/kg body weight to induce diabetes. Mice with whole blood glucose levels remaining at >360 mg/dL for more than 2 weeks were used as diabetic recipients. Three to 5 mice were housed in each cage and fed standard pelleted food and tap water ad libitum. The animal room had an automatic lighting cycle with 12 h of light and 12 h of darkness. The animals were treated humanely in accordance with the laboratory animal guidelines of Chang-Gung Memorial Hospital.

### 2.2. Islet Isolation

Pancreatic islets were isolated from C57BL/6 mice by collagenase digestion, enriched on a histopaque density gradient, and finally hand picked. Briefly, after administering sodium amobarbital to the mice to induce anaesthesia, we distended the pancreas of each nonfasted healthy mouse with 2.5 mL RPMI-1640 medium containing 1.5 mg/mL collagenase and then excised and incubated these in a 37°C water bath. The islets were purified using a density gradient and were hand-picked under a dissecting microscope. Isolated islets with diameters of 150 *μ*m were collected and separated into groups of 50 islets per group. To minimize the influence of batch-to-batch variation in islet function on the experimental observations, each batch of islets isolated from 8–10 mice in a single day were separated into equal groups and transplanted into an equal number of mice in both the control and experimental groups on the same day.

### 2.3. Renal Subcapsular Transplantation

The islets were centrifuged (500 g for 90 seconds) in a PE-50 tube connected to a 200 *μ*L pipette tip. With the recipient mouse under amobarbital anaesthesia, the left kidney was exposed through a lumbar incision. A capsulotomy was performed in the lower pole of the kidney, and the tip of the PE-50 tube (Clay Adams, Parsippany, NJ, USA) was advanced beneath the capsule toward the upper pole, where the islet graft was implanted using a Hamilton syringe. The capsule was left unsutured. The retroperitoneal cavity was closed using a 2-layered suture.

### 2.4. Targeting and Nontargeting Lentiviral Transduction to Silence p27^kip1^ in Islets

We transduced islets with small hairpin RNAs (shRNAs) to silence p27^kip1^ and used nontarget shRNAs as controls and then examined the effect of p27^kip1^ silencing on the adaptation of adult mice islets. Briefly, freshly isolated islets, at a concentration of 50 islets per well, were plated in a 96-well tissue culture plate with 150 *μ*L of RPMI-1640 medium containing 8 *μ*g/mL of polybrene. Lentivirus was then incubated with islets at 20 multiplicity of infection (MOI) for 24 h at 37°C. The next day, the islets were transferred to new plates, washed twice, and further cultured at 37°C, with the medium being changed on a daily basis. At 72 h following isolation, the islets were collected for transplantation. p27^kip1^ gene silencing was performed by infecting the islets with 4 clones of MISSION shRNA Lentiviral Particles (Sigma, Saint Louis, MO, USA) that are designed to target cyclin-dependent kinase inhibitor 1B (p27^kip1^). These 4 clones were TRCN0000071063, (CCGGCGCAAGTGGAATTTCGACTTTCTCGAGAAAGTCGAAATTCCACTTGCGTTTTTG); TRCN0000071064, (CCGGCCGGCATTTGGTGGACCAAATCTCGAGATTTGGTCCACCAAATGCCGGTTTTTG); TRCN0000071066, (CCGGCCTTTAATTGGGTCTCAGGCACTCGAGTGCCTGAGACCCAATTAAAGGTTTTTG); TRCN0000071067, CCGGCCCGGTCAATCATGAAGAACTCTCGAGAGTTCTTCATGATTGACCGGGTTTTTG.

### 2.5. The Effect of p27^kip1^ Silencing on Islet Cell Cycle Protein In Vitro

 At 24 hours of transduction, equal batches of targeting and non-targeting islets (about 300 islets per batch) were washed 3 times with ice-cold phosphate-buffered saline and lysed with a buffer containing 150 mmol/L NaCl, 0.2 mmol/L ethylenediamine-tetraacetic acid 20 mmol/L Tris-HCl (pH 7.4), and 0.5% sodium dodecyl sulfate supplemented with complete protease inhibitor cocktail (Roche Diagnostic Deutschland GmbH, Mannheim, Germany). After 20-minute incubation at 4°C, the lysate was centrifuged at 13,000 ×g for 15 minutes at 4°C, and the supernatant was collected as the total islet protein content. Total islet proteins separated on a 12% sodium dodecyl sulfate polyacrylamide gel were electrophoretically transferred onto nitrocellulose membranes. After Ponceau S staining to check transfer efficiency, the membrane was blocked for 2 hours at room temperature with 10% nonfat dry milk (NFDM) in Tris-buffered saline (pH 7.4) containing 0.1% Tween-20 (TBST). It was then incubated overnight at 4°C in NFDM-TBST containing 2 *μ*g/mL each of antibodies against *β*-actin (housekeeping), p27^kip1^ (G1/G0 checkpoint), cyclin D1 (G1/S), cyclin B1 (G2/M), and FoxM1, washed 3 times with TBST, and incubated for 1.5 h at room temperature with horseradish peroxidase-conjugated anti-rabbit immunoglobulin G antibody (0.1 *μ*g/mL NFDM-TBST). After 3 washes with TBST, the bound antibodies were detected by using the VisGlow chemiluminescent kit obtained from Visual Protein Biotechnology (Taipei, Taiwan) and Kodak BioMax MS films. The cell cycle proteins were expressed as the ratio of band density of each protein over that of *β*-actin in the same batch of total islet proteins.

### 2.6. The Effect of p27^kip1^ Silencing on Islet Glucose-Stimulation Insulin Secretion In Vitro

Function of islets transduced with shRNAs to silence p27^kip1^ or the non-target shRNA control were evaluated by glucose-stimulation insulin secretion. After 24 h of transduction and at 3 and 7 days of culturing, batches of 30 islets per well were sequentially exposed to different concentrations of glucose. Islets were incubated in 100 mg/dL glucose for 60 min. After the medium was collected, the same batch of islets was then stimulated with 360 mg/dL of glucose for 60 min. The net insulin secretion from the glucose stimulation test (GSIS) was calculated as the difference of the insulin content between the medium containing 360 mg/dL and medium containing 100 mg/dL glucose. The stimulation index (SI) was considered as the ratio of insulin content of the medium containing 360 mg/dL of glucose over that of the medium containing 100 mg/dL of glucose for the same batch of islets.

### 2.7. The Short-Term *Ex Vivo* Effects of p27^kip1^ Silencing on Islets Cell Replication and Function

To study the short-term effect of p27^kip1^ silencing on islet cell function and replication, mouse islets transduced with shRNAs to p27^kip1^or the non-target shRNA control were transplanted to both normoglycemic healthy mice and mice with streptozotocin-induced diabetes. Whole blood glucose and body weight were measured every other day. Six days after transplantation, the islet grafts were removed for immunohistochemical examinations. The relative number of cells in the islet graft undergoing replication was estimated by the relative numbers of cells with nuclei staining positive for Ki67 for an average of 250 cells per graft (*n* = 4 per group).

### 2.8. The Long-Term *Ex Vivo* Effects of p27^kip1^ Silencing on Islets Cell Function

 To study the long-term effects of p27 silencing on the function of islet grafts, mouse islets transduced with shRNAs to p27^kip1^or the non-target shRNA control were transplanted to mice with streptozotocin-induced diabetes 24 hours after transduction and 48 hours after incubation in the culture medium. We transplanted 200 islets per recipient to study the effect of p27 silencing on islet function *in vivo*. After transplantation, the body weight and whole blood glucose level in blood samples drawn from a tail vein were measured 2 to 3 times a week for 12 weeks, and the duration of temporary hyperglycaemia was considered as the period of time between transplantation and 2 consecutive measurements of whole blood glucose level below 200 mg/dL. At the end of the 12-week observation period, left nephrectomy was performed on all mice to remove the islet graft. All nephrectomized mice were kept alive for 2 weeks to confirm graft function, and then, the pancreas was removed for measuring the insulin content.

### 2.9. Measurement of Insulin Content

 Insulin content in the pancreas remnant and the islet graft was determined by using the acid-ethanol method. Briefly, the pancreas or graft-bearing kidney from nonfasted mice was removed randomly between 8 and 10 AM on the indicated date, homogenized in an acid-ethanol solution, and stored overnight at 4°C. After centrifugation at 2400 rpm for 30 min, the supernatant was collected and stored at −20°C. The pancreas remnant and islet grafts were then homogenized again in a fresh aliquot of acid-ethanol solution and the insulin was re-extracted overnight. After centrifugation, the supernatant was collected and pooled with the first extracted sample. Finally, the insulin concentration was measured using radioimmunoassay.

### 2.10. Statistical Analysis

 Data are expressed as means ± standard error. Statistical differences between means were analyzed using a paired or unpaired Student's *t*-test, as appropriate. The cumulative cure rate in groups of the long-term effect study was assessed using the Kaplan-Meier method. The log rank test was used to analyze differences in the cure rates between groups of mice. A value of *P* < 0.05 was considered significant.

## 3. Results

**Figure 1:**
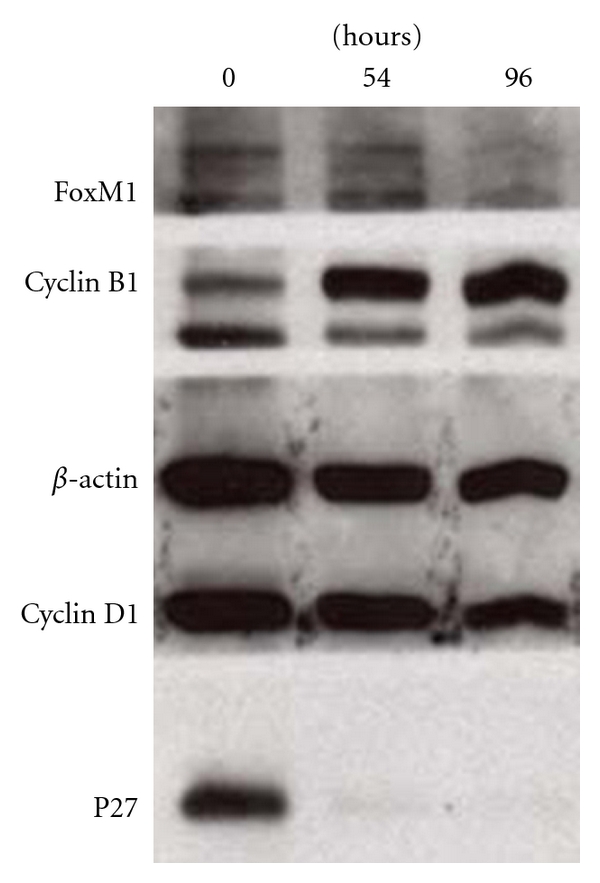
Effect of p27^kip1^ silencing on the expression of islet cell cycle proteins. Total islet cell protein contents of targeting islets were prepared right after isolation (0 hr) and at 54 and 96 hours (hrs) after viral infection; separated on a 12% SDS gel and electrophoretically transferred onto nitrocellulose membranes. After checking transfer efficiency, the membrane was blocked and incubated overnight in buffer containing 2 *μ*g/mL each of rabbit antibodies against mouse p27^kip1^, cyclin D1, *β*-actin, cyclin B1, and FoxM1; incubated with horseradish peroxidase-conjugated goat anti-rabbit immunoglobulin G antibody. The bound antibodies were detected by using chemiluminescent kit and film development. The molecular weight is 27 kD for p27, 36 kD for cyclin D1, 43 kD for *β*-actin, 62 kD for cyclin B1, and 84 kD for FoxM1.

Table 1 reveals that the lentivirus carrying p27^Kip1^-targeting shRNA effectively decreased the expression of p27^Kip1^ protein in adult mouse islets at 54 and 96 h after infection and a representative western blot was shown in Figure 1. The p27^Kip1^ protein content in cultured islets at 54 hours after viral infection was only 22% that in freshly isolated islets, and the suppression of p27^Kip1^ protein was maintained even after 96 hours of infection. Table 1 shows that the suppression of p27^Kip1^ expression was accompanied by the increment in the levels of B1 and FoxM1 proteins, which suggest that the reduced p27^Kip1^ expression allows islets cells to enter the cell cycle. The control islets infected with the non-targeting lentivirus had a p27^Kip1^/actin ratio of 0.56 ± 0.11 (*n* = 6), 0.80 ± 0.18 (*n* = 4), and 0.95 ± 0.24 (*n* = 4) at 24, 54, and 96 hours after infection, respectively, in comparison with the ratios obtained for the freshly isolated islets.

To study the effect of lentivirus transfection on islets function, the glucose-stimulated insulin secretion (GSIS) and stimulation index (SI) of islets were measured at 3 and 7 days after targeting and non-targeting viral infection.  Table 2 shows that the ratio and net difference of insulin secretion between 360 mg/dL and 100 mg/dL glucose of cultured islets with targeting did not differ from that of islets with non-targeting viral infection.

**Figure 2:**
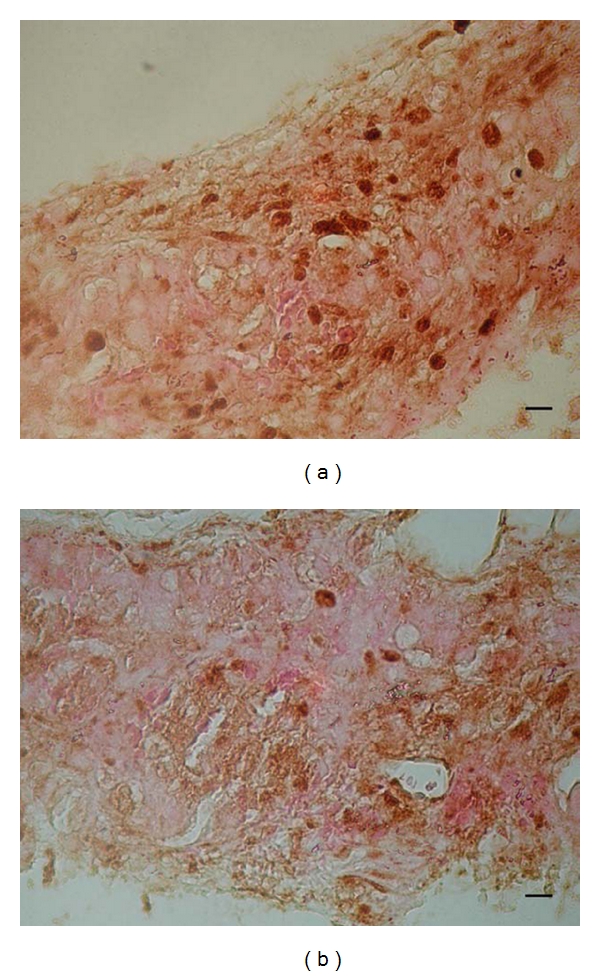
Short-term *ex vivo* effect of p27^Kip1^ silencing on islet cell proliferation in diabetic syngeneic recipients. Batches of 50 of targeting (a) or non-targeting (b) transduced islets were implanted in the subcapsular space of the left kidney of B6 mice with streptozotocin-induced diabetes, and grafts were removed 6 days later for immunohistochemistry by using the anti-Ki67 antibody. The deep-colored oval structures represent positively stained nuclei. The black scale bar indicates a length of 10 micrometers.

To investigate the short-term effect of p27^Kip1^ silencing on islet cell proliferation, batches of 50 each of targeting and non-targeting transduced islets were implanted in the subcapsular space of the left kidney of normal healthy mice and B6 mice with streptozotocin-induced diabetes; 6 days later, the grafts were removed and immunohistochemistry was performed. When the mice with streptozotocin-induced diabetes were used as recipients, the number of nuclei staining positive for Ki67 was 0.35 ± 0.12 and 0.12 ± 0.07 (*n* = 4, *P* < 0.05) for grafts of targeting and non-targeting islets, respectively.  Figure 2 shows the typical results of immunohistochemistry of islets grafts of the targeting group (Figure 2(a)) and the non-targeting group (Figure 2(b)). In normal healthy mice, anti-Ki67 antibody staining was hardly detectable in the nuclei of islet graft cells from both the targeting and non-targeting groups (data not shown). For targeting and non-targeting mice in the diabetic recipient groups, the whole blood glucose level was 373 ± 16 mg/dL and 360 ± 6 mg/dL (*n* = 4, *P* > 0.05) at day 0 and 412 ± 20 mg/dL and 429 ± 46 mg/dL (*n* = 4, *P* > 0.05) at day 6 after implantation, respectively. The body weight for targeting and non-targeting mice in the diabetic recipient groups was 23.0 ± 0.8 g and 23.3 ± 1.3 g (*n* = 4, *P* > 0.05) at day 0 and 24.6 ± 0.3 g and 24.2 ± 1.0 g (*n* = 4, *P* > 0.05) at day 6 after transplantation respectively.

**Figure 3:**
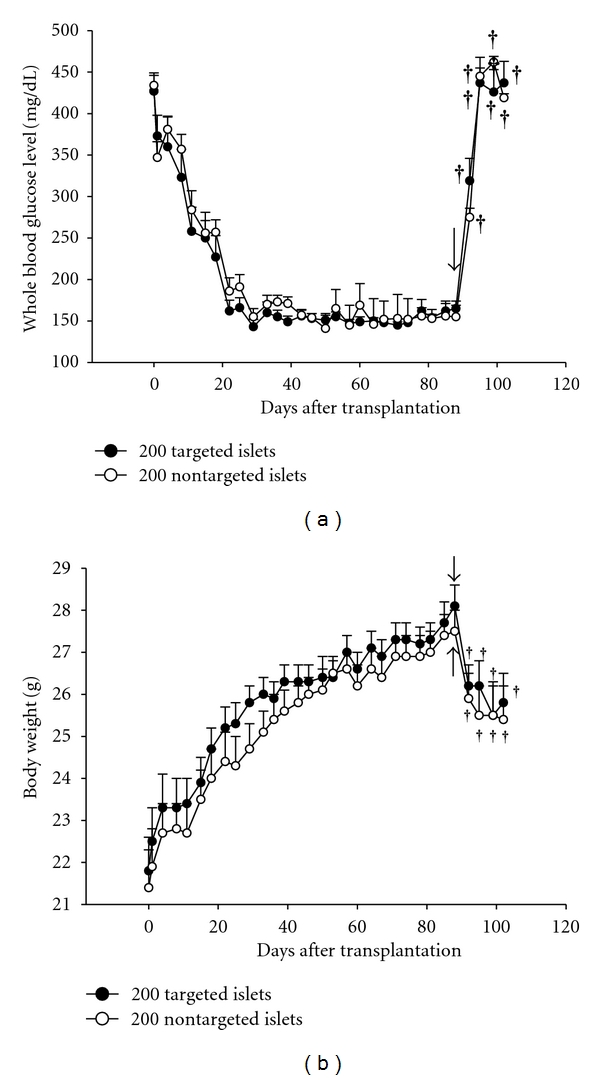
Long-term *ex vivo* effect of p27^Kip1^ silencing on the blood glucose level (a) and body weight (b) in a syngeneic mouse transplantation model. Each mouse with streptozotocin-induced diabetes was transplanted with 200 mouse islets transduced with either shRNAs to p27^kip1^or the non-target shRNA control under the left renal capsule at day 0. After transplantation, blood glucose level and body weight were measured. At 84 days following transplantation, the graft-bearing kidneys of all mice were removed by nephrectomy (solid arrow), and all mice were kept alive for 2 weeks before being sacrificed. In Figures 3(a) and 2(b), *P < 0.05, ^†^ *P* < 0.01 indicates the marked mean compared to that on day 84 in the same group using paired Student's *t*-test.

**Figure 4:**
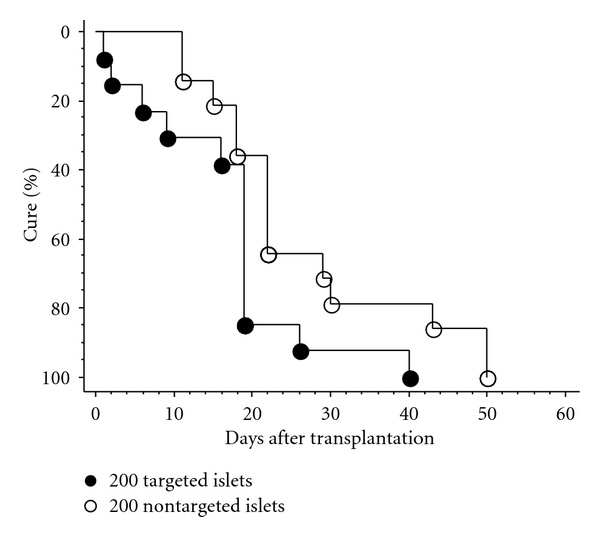
Curves of cumulative diabetes mellitus cure rate for syngeneic islet transplantation. Each streptozotocin-induced diabetic mouse was transplanted with 200 mouse islets transduced with either shRNAs to p27^kip1^ or the non-target shRNA control implanted underneath the left renal capsule at day 0. Cure of diabetes was defined as the stable restoration of normoglycemia (<200 mg/dL glucose). Percent cure 50 (%) stands for half number of the transplanted diabetic mice becoming normoglycemia. The beneficial effect of p27^kip1^ silencing on the function of islet grafts was reflected by the high cumulative cure rate of diabetes. The difference between two groups was analyzed with the log rank test. The value of the comparison for targeting versus non-targeting was *P* < 0.05.

To study the long-term effect of p27^Kip1^ silencing on islet graft function, batches of 200 isogeneic islets with targeting or non-targeting transduction were implanted in the subcapsular space of the left kidney of B6 mice with streptozotocin-induced diabetes, and the whole blood glucose and body weight were measured. As shown in Figures 3(a) and 4, all treated diabetic mice that received 200 islets converted to normoglycemia, and the mice in the targeting group had significantly shorter temporary hyperglycaemia period than mice in the non-targeting group (16.5 ± 2.9, *n* = 13 versus 25.9 ± 3.5, *n* = 14, *P* < 0.05 in Table 3). The long-term *ex vivo* beneficial effect of p27^Kip1^ silencing on graft function was also indicated by the significantly higher cumulative cure rate for diabetes in mice receiving 200 targeting islets than that in mice receiving 200 non-targeting islets (*P* < 0.05) (Figure 4). At the end of the 12-week observation period, the grafts were removed to confirm the hypoglycaemic effect. Three days after the removal of the graft-bearing kidney, the mice showed rapid elevation of whole blood glucose level and a significant decrease in body weight (Figures 3(a) and 3(b)). There was no difference between the targeting and non-targeting groups in terms of insulin contents of the graft and pancreatic remnant at 12 weeks after transplantation (Table 3).

## 4. Discussion

In this study, we transduced adult islets with lentivirus-carrying shRNA to silence 80% of p27^Kip1^ protein, and the resultant suppression of p27^Kip1^ expression lasted for over 96 hours after infection. The transduction and the subsequent p27^Kip1^ suppression did not influence islet functions in terms of glucose-stimulated insulin secretion. It has been demonstrated that suboptimal number of islet tissue was insufficient to achieve normoglycemia in diabetic recipient; graft beta cell replication was increased initially but not by 18 days and after despite persistent hyperglycemia, and beta cell mass fell progressively [16]. Therefore, in this study, 50 islet tissues per recipient are used for short-term transplantation experiment to render islet beta cell a higher replication rate in diabetic recipient; 200 islet tissue is used for long-term experiment in order to shorten the temporary hyperglycemia days and avoid falling beta cell mass by persistent hyperglycemia. In the short-term *ex vivo* study, when normoglycemic healthy mice were used as recipients, neither control islets nor islets with p27^Kip1^ silencing had detectable cells with nuclei staining positive for Ki67, at 6 days after transplantation. On the contrary, adult islets with p27^Kip1^ silencing expressed more nuclei and higher density of positive Ki67 staining than the control islets, at 6 days after the islets were transplanted to mice with streptozotocin-induced diabetes. Our data suggest that p27^Kip1^ suppression alone does not enhance islet cell proliferation in normoglycemic mice but hyperglycemia and persistent p27^Kip1^ suppression have a synergistic effect on islet cell replication in terms of nuclei staining positive for Ki67. To further confirm the complementary effect of hyperglycemia and p27^Kip1^ silencing on beta cell replication, we transplanted adult mouse islets with or without p27^Kip1^ silencing to diabetic mice and followed up all treated mice for 3 months. The beneficial effect of p27^Kip1^ silencing on graft replication was indicated by the significantly shorter temporary hyperglycemic period and the significantly higher cumulative cure rate for diabetes in mice receiving targeting islets than that of mice receiving the same number of non-targeting islets.

During the early G1 phase of the cell cycle, cyclin D-cyclin dependent kinase 4/6 catalyzes phosphorylation of retinoblastoma protein (pRB) and the phosphorylated pRB inhibits anaphase-promoting complex- (APC/C-) mediated polyubiquitination and subsequent proteolysis of skp2, which results in increase of skp2 and decrease of p27^Kip1^ protein [17]. The degradation of p27^Kip1^ is essential for cells to enter G1/S phases for replication and the resultant adaptive expansion of pancreatic beta cells [11, 12]. During S-G2-M phases of each beta cell division, glucose downregulates cyclin D2 expression that will reduce phosphorylated pRB and cause decrease of skp2 and increase of p27^Kip1^ protein [10, 17]. The increment of p27^Kip1^ protein during S-G2-M phases of each beta cell division prevents cells from immaturely entering the next cycle [17]. In late G2 phase, the amount of B1 protein in Xenopus oocytes is 20- to 30-fold higher than in G1 and a threshold level of cyclin B1 protein must be reached before mitosis can proceed [18]. Once initiated, progression through mitosis is dependent on the degradation of several cell cycle regulatory proteins by APC/C, including cyclin B1 and skp2, which again prevents cells inappropriately entering other phases during mitosis [19, 20]. Our previous study revealed that isolated islets of adult mice persistently express high level of cyclin B1 especially when the culture medium contains high glucose [14]. In this study, we used shRNAs to silence p27^Kip1^ and used hyperglycemia as a complementary factor to examine the synergistic effect of glucose and p27^Kip1^ on the adaptation of adult mice islets. Although the mechanism of action of the synergistic effect of hyperglycemia and p27^Kip1^ silencing on adult islet beta cell replication is not yet clear, we hypothesize that the persistent suppression of p27^Kip1^ by lentivirus-carrying shRNA on adult islet cells will drive more cells into the G1/S phase and the high glucose-mediated persistent elevation of cyclin B1 will prepare more cells to be ready to enter mitosis and increase beta cell replication.

In conclusion, our data suggested that adult mouse islet beta cells can replicate when the metabolic demands increase and there is a synergistic effect of hyperglycemia and concurrent suppression of p27^Kip1^ on islet beta cell replication.

## Figures and Tables

**Table 1:** Effect of p27^Kip1^ silencing on the expression of cell cycle proteins in adult mouse islets. Cellular lysates of freshly isolated mouse islets and cultured islets at 54 and 96 h after infection with lentivirus to silence p27^Kip1^ were separated on 12% sodium dodecyl sulfate-polyacrylamide gel and electrophoretically transferred onto a nitrocellulose membrane, which was then incubated with antibodies against *β*-actin, p27^Kip1^, cyclin B1, cyclin D1, and FoxM1. Cellular lysates of control islets were collected at 24, 54, and 96 h after infection with virus carrying non-target shRNAs for the determination of p27^Kip1^ protein level. Bound antibodies were detected by using a chemiluminescence kit. The levels of cell cycle proteins were expressed as the ratio of the band density of each protein over that of *β*-actin of the same batch of islet proteins. The protein content of freshly isolated islets was used as the standard for comparison to express the changes in the levels of cell cycle proteins after virus infection. Data were expressed as mean ± standard error. ^a^ *P* < 0.05, ^b^ *P* < 0.01, ^c^ *P* < 0.005 when the indicated mean was analyzed using the single *t*-test.

Hours after viral infection	p27^Kip1^/actin non-targeting control	p27^Kip1^/actin targeting	B1/actin targeting	D1/actin targeting	FoxM1/actin targeting
0	1	1	1	1	1
24	0.56 ± 0.11^a^, *n* = 6				
54	0.80 ± 0.18, *n* = 4	0.22 ± 0.06^b^, *n* = 4	9.40 ± 2.42^a^, *n* = 4	0.97 ± 0.11^a^, *n* = 4	1.31 ± 0.10^b^, *n* = 4
96	0.95 ± 0.24, *n* = 4	0.19 ± 0.03^c^, *n* = 4	3.26 ± 0.85^a^, *n* = 4	0.93 ± 0.12^b^, *n* = 4	1.88 ± 0.46^a^, *n* = 4

**Table 2:** The effect of p27^kip1^ silencing on islet glucose-stimulation insulin secretion *in vitro*. After 24 h of transduction and at 3 and 7 days of culturing, batches of 30 islets per well were sequentially exposed to different concentrations of glucose. Islets were incubated in 100 mg/dL glucose for 60 min. After the medium was collected, the same batch of islets was washed and then stimulated with 360 mg/dL of glucose for 60 min. The net difference of insulin secretion from the glucose stimulation test (GSIS; ng/islet × 60 min) was calculated as the difference of the insulin content between the medium containing 360 mg/dL and medium containing 100 mg/dL glucose. The stimulation index (SI) was considered as the ratio of insulin content of the medium containing 360 mg/dL of glucose over that of the medium containing 100 mg/dL of glucose for the same batch of islets.

	Time	Targeting islet	Non-targeting islet control	*P* value
GSIS (ng/islet × 60 min)	Day 3	0.17 ± 0.02 (*n* = 6)	0.20 ± 0.02 (*n* = 10)	>0.05
	Day 7	0.24 ± 0.09 (*n* = 6)	0.17 ± 0.02 (*n* = 12)	>0.05
SI	Day 3	2.6 ± 0.3 (*n* = 6)	2.8 ± 0.4 (*n* = 10)	>0.05
	Day 7	2.4 ± 0.3 (*n* = 6)	2.6 ± 0.3 (*n* = 12)	>0.05

**Table 3:** Effect of p27^Kip1^ silencing on the period of temporary hyperglycaemia and insulin contents of islet graft and pancreatic remnant. Batches of 200 isogeneic islets with targeting and non-targeting (control) transduction were implanted in the subcapsular space of the left kidney of B6 mice with streptozotocin-induced diabetes. After transplantation, blood glucose was measured, and the duration of temporary hyperglycemia was the period between transplantation and the stable restoration of normoglycemia (<200 mg/dL). The graft-bearing kidneys were removed 12 weeks after transplantation for the measurement of insulin contents. The pancreatic remnants were removed 2 weeks after nephrectomy for measuring the insulin content. ^a^ *P* < 0.05 when the indicated means were analyzed using unpaired Student's *t*-test.

No. of Islets	Lentivirus	*N*	Period of temporary hyperglycaemia (days)	GIC (*μ*g)	PIC (*μ*g)
200	Targeting	13	16.5 ± 2.9^a^	1.75 ± 0.18	0.22 ± 0.07
200	Control	14	25.9 ± 3.5^a^	1.54 ± 0.17	0.22 ± 0.05
